# Interplay of ortho*-* with spiro-cyclisation during iminyl radical closures onto arenes and heteroarenes

**DOI:** 10.3762/bjoc.9.120

**Published:** 2013-06-04

**Authors:** Roy T McBurney, John C Walton

**Affiliations:** 1 EaStCHEM School of Chemistry, University of St. Andrews, St. Andrews, Fife, KY16 9ST, UK

**Keywords:** cyclisation, EPR spectroscopy, free radicals, heterocycles, oxime carbonates

## Abstract

Sensitised photolyses of ethoxycarbonyl oximes of aromatic and heteroaromatic ketones yielded iminyl radicals, which were characterised by EPR spectroscopy. Iminyls with suitably placed arene or heteroarene acceptors underwent cyclisations yielding phenanthridine-type products from ortho-additions. For benzofuran and benzothiophene acceptors, spiro-cyclisation predominated at low temperatures, but thermodynamic control ensured ortho-products, benzofuro- or benzothieno-isoquinolines, formed at higher temperatures. Estimates by steady-state kinetic EPR established that iminyl radical cyclisations onto aromatics took place about an order of magnitude more slowly than prototypical C-centred radicals. The cyclisation energetics were investigated by DFT computations, which gave insights into factors influencing the two cyclisation modes.

## Introduction

Radical cyclisations onto aromatic acceptors take place readily, even though disruption of the 6π-electron system necessarily occurs. The most commonly encountered type is C-centred radical addition (often an aryl radical) to an aromatic or heteroaromatic ring ortho to the point of attachment of the tether. In Pschorr and related processes re-aromatisation follows with production of phenanthrene-type derivatives [1–2]. Spiro-cyclisations in which tethered radicals add to the ipso-C-atoms of the rings are less common, although minor spiro*-*products not infrequently accompany the main ortho*-*ones in Pschorr syntheses [3–5]. Cyclisations onto arenes by N-centred radicals are rarer, but iminyl radical ArC(R)=N^•^ closures are well documented. Forrester and co-workers were probably the first to utilise iminyl radicals synthetically. They obtained iminyls by persulfate oxidation of imino-oxyacetic acids in aqueous solvents and prepared azines [6], *N*-heterocycles [7–8], and other derivatives [9]. This research initiated spiralling interest by synthetic chemists in iminyl radical-mediated preparations. Recently iminyls have been generated from quite a variety of precursors [10–15], and their cyclisations onto arenes [16–21] and heteroarenes [22–24] have attracted attention. Iminyl cyclisations have also been utilised in natural-product syntheses [10,16,25–26]. Iminyl radical spiro-cyclisations onto aromatics have been reported in a few cases, and spiro-intermediates have occasionally been proposed in mechanistic explanations [15,18,27–29].

Although a moderate amount of information about iminyl radical structure and reactivity exists, few conceptual tools to help predict their cyclisation selectivity are available. EPR spectroscopic and other evidence established that iminyl radicals behave as σ-type species with their unpaired electrons in orbitals in the nodal plane of their C=N π-systems [30–32]. This precludes substantial delocalization of the unpaired electron into the ring π-system of aryliminyls. Consequently, strong effects from ring substituents of aryliminyls are not expected to come into play. Small to moderate size iminyl radicals terminate rapidly at diffusion-controlled rates by N–N coupling to give azines [32]. β-Scission reactions yielding nitriles do occur, but are not important at *T* < ~420 K for aryliminyls or for iminyls with primary alkyl substituents [32]. The rate constants for H-abstraction by iminyls yielding imines are more than an order of magnitude slower than for C*-*centred analogues [33]. Iminyls undergo 5-*exo*-ring closures onto alkenes about a factor of 25 more slowly than C*-*centred analogues [34]. Since ring closure is often in competition with H-abstraction, the comparatively slow H-abstraction by iminyls is important for the success of many heterocycle syntheses.

Spiro-cyclisations of iminyls lead to formation of strained quaternary C-atoms, and the spiro-intermediates have no straightforward reaction channel for return to aromaticity. The process might be reversible, depending on the architecture of the chain and the extent of strain in the spiro*-*radical. On the other hand ortho-cyclisations can easily be followed by return to aromaticity of the cyclohexadienyl radicals, either by transfer out of the labile H-atom, or by transfer of an electron to a suitable sink with generation of the corresponding carbocation, followed by proton loss. Kinetic or other data to help predict which mode would be favoured for a novel iminyl radical is essentially nonexistent.

**Figure 1:**
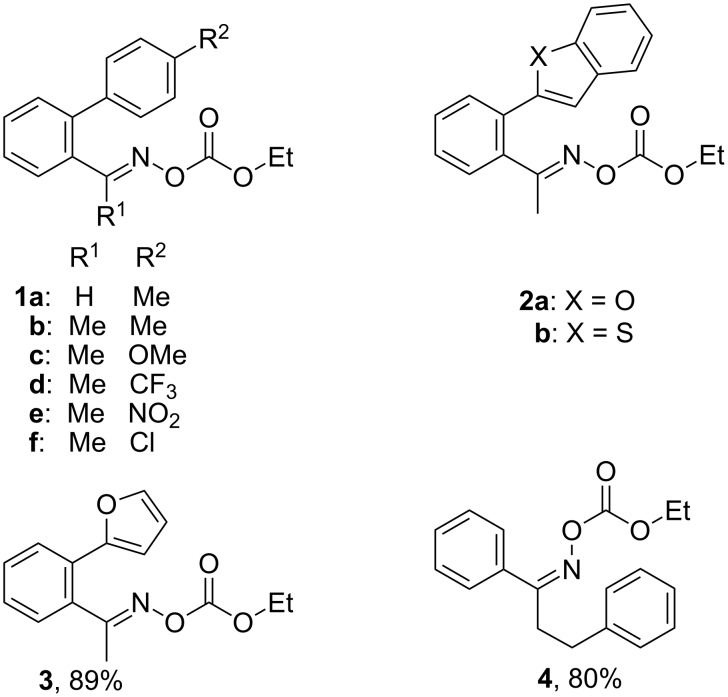
*O*-Ethoxycarbonyl oximes prepared.

We discovered recently that oxime carbonates ArC(R)=N–OC(O)OR’ are clean and convenient precursors for iminyl as well as O-centred radicals [26,35]. Their weak N–O bonds selectively cleave on UV photolysis, particularly when sensitised with 4-methoxyacetophenone (MAP), thus facilitating investigations of the behaviour of both iminyl and alkoxycarbonyloxyl radicals R’OC(O)O^•^. A distinct advantage of these precursors is that they enable the iminyl radical intermediates to be directly monitored by EPR spectroscopy. We have now prepared a representative set of oxime carbonates with the aim of studying competition between ortho- and spiro-ring closures of the released iminyl radicals. Precursors **1a–f**, **2a**,**b**, **3** and **4**, consist of *O*-ethoxycarbonyl derivatives of oximes with various aromatic and heteroaromatic architectures (Figure 1). Compounds **1a–f** contain comparatively rigid arms and their aromatic acceptors range from electron-withdrawing to electron-releasing in character. In compounds **2a**,**b** and **3** heteroarenes replace the benzene rings and in **4** the arm is much more flexible.

This paper reports our study of the chemistry of these compounds by means of product analyses, solution EPR spectroscopy and DFT computations. We encountered an intriguing interplay between spiro-cyclisation and ortho-cyclisation of the iminyl intermediates and factors affecting this are weighed up.

## Results and Discussion

The arene and heteroarene-based oxime carbonates **1** and **2** were prepared as described previously by reaction of the corresponding aromatic ethanone oximes with ethyl chloroformate [26]. Precursors **3** and **4** were made in a similar way from the oximes of 2-furanylphenylethanone and 1,3-diphenylpropan-1-one.

### Ring closures of aryl-iminyl radicals

**Scheme 1:**
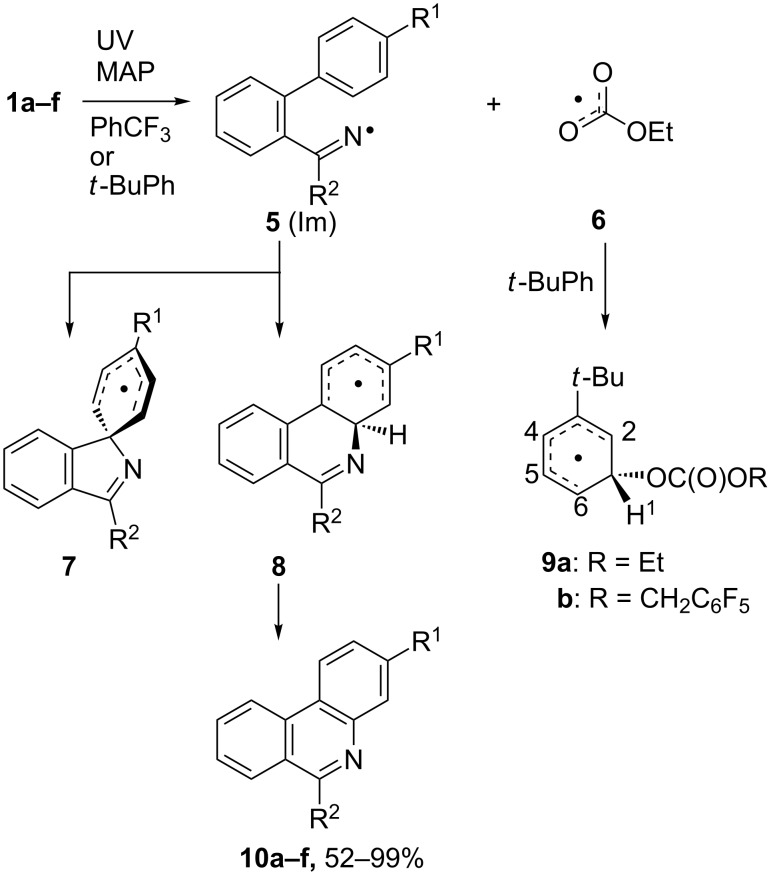
Photochemical reactions of biphenyl oxime carbonates.

Individual members of the set of substituted biphenyl *O*-ethoxycarbonyl oximes **1a–f** were UV irradiated for 3 h at ambient temperature in deoxygenated benzotrifluoride solutions with 1 equiv wt/wt of MAP as a photosensitizer. As communicated previously, 3-substituted phenanthridines **10a–f** were isolated in good to quantitative yields (52–99%, Scheme 1) irrespective of the nature of the 4-substituent [26]. No spiro-products were detected even by GC–MS analyses of reaction mixtures. Byproducts included traces of imines ArC(R^2^)=NH (ImH) and the ketones ArC(R^2^)=O from imine hydrolyses. Photolysis of the more flexible precursor **4** gave a complex mixture of products. The MS and NMR data indicated that the main components were probably the corresponding imine ImH and ketone together with the iminyl radical dimer (Im_2_). Neither spiro- nor ortho-cyclised products had formed. Therefore, for **4** in PhCF_3_ solvent, iminyl radical ring closures were too slow to compete with H-atom abstractions and terminations.

**Figure 2:**
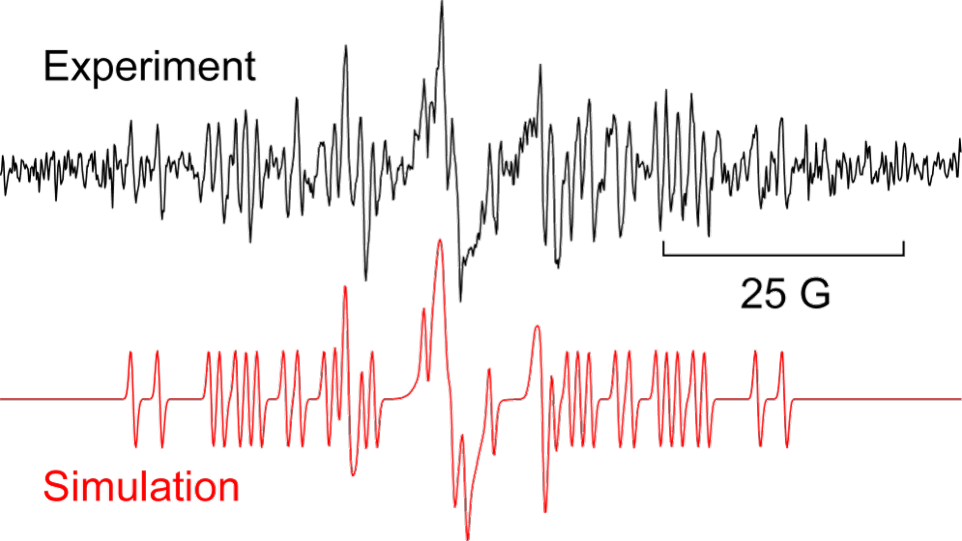
EPR spectrum during photolysis of **1f** in *t*-BuPh at 240 K. Top (black): experimental spectrum. Bottom (red): computer simulation.

The photolytic reactions of oxime carbonates **1a–f** and **4** were next investigated by 9 GHz EPR spectroscopy. Deaerated samples of each oxime carbonate, plus 1 equiv of MAP, in *t*-BuPh or cyclopropane solvent, were irradiated with a 500 W unfiltered Hg lamp directly in the spectrometer resonant cavity. The spectrum obtained from precursor **1f** (Figure 2) shows a central 1:1:1 triplet from iminyl radical **5f** together with a second species. Similar spectra were obtained, in the temperature range 210 to 270 K, from all the other members of the set, including **4**, showing the corresponding iminyl radicals plus the same second radical. The EPR parameters of all the iminyls were very similar [*g* = 2.0030¸ *a*(N) = 10.0 G] and closely in line with literature data for ArCMe=N^•^ type radicals [36–37].

Simulation of the second species indicated one large and four smaller doublet hyperfine splittings (hfs) characteristic of a cyclohexadienyl type radical (Table 1). This spectrum was evidently due to the intermediate from addition of some radical meta to the *tert*-butyl substituent of the solvent. It is known that ArCMe=N^•^ type radicals do not add to *t*-BuPh under EPR conditions [19,37] neither do EtO^•^ radicals (from dissociation of **6**), and hence, we assign this spectrum to the ethoxycarbonyloxyl adduct **9a**. This identification was supported by a DFT computation [38] that gave hfs in close agreement with experiment (Table 1). This was a surprising result because previously the only radicals of type **9** that had been spectroscopically detected had resulted from additions of phenyl [34] or bridgehead radicals (bicyclo[2.2.2]oct-1-yl and adamantyl) [39]. These are localized σ-type radicals with bent or pyramidal centres and significant s-character. Ethoxycarbonyloxyl (**6**) is planar with a SOMO delocalized over the whole OC(O)O unit. The observation of **9a** at temperatures below 273 K is dramatic evidence of the exceptionally high reactivity of alkoxycarbonyloxyl radicals. An interesting feature was that addition was selective for meta to the *t*-Bu group; as was previously observed with the σ-radicals. Product studies with bridgehead radicals at higher temperatures (80 °C) had also revealed this preference for meta-attack [40–41]. The selectivity for meta-addition may result from the electron-releasing character of the *t*-Bu substituent. The SOMO in **9a** has a node at C(3) so electron–electron repulsion is smaller than in the SOMOs for para*-* or ortho-attack. This will lower the activation energy for meta-addition relative to para*-* or ortho*-*addition.

**Table 1:** EPR parameters of cyclohexadienyl radicals **9** from meta*-*additions to *t*-BuPh^a^.

Radical	T/K or method	*g*-factor	*a*(H^1^)	*a*(H^2^)	*a*(H^4^)	*a*(H^5^)	*a*(H^6^)

**9a**	240	2.0025	34.6	8.1	13.1	2.8	9.2
**9a**	DFT^b^	–	35.0	−8.3	−12.8	3.5	−9.8
**9b**^c^	210	2.0026	33.5	8.1	13.1	2.7	9.3
**9(Ph)**^d^	220	2.0030	35.5	8.1	13.3	2.7	9.1
**9(222)**^e^	220	2.0030	42.6	8.1	13.1	2.8	9.0

^a^At 9.4 GHz in *t*-BuPh solution; hfs in Gauss. Note that the signs of hfs cannot be obtained from isotropic EPR spectra. ^b^UB3LYP/6-311+G(2d,p); hfs computed with the epr-iii basis set [42] designed for EPR hfs, were virtually identical. ^c^R. T. McBurney and J. C. Walton unpublished. ^d^As **9** but with Ph in place of OC(O)OEt [34]. ^e^As **9** but with bicyclo[2.2.2]oct-1-yl in place of OC(O)OEt [39].

For all precursors **1a–f** the EPR spectra revealed uncyclised iminyls (**5a–f**) together with **9** up to *T* ~270 K. Above this temperature the EPR spectra became too weak for radical identification. No cyclohexadienyl type radicals from either spiro or ortho ring closure (**7** or **8**) were detected. It can be concluded that the iminyl cyclisations are comparatively slow and, based on the previous product analyses, the ortho- (Ar_1_-6) mode predominates at room temperature and above.

### Spiro-cyclisations with benzofuran and benzothiophene acceptors

**Figure 3:**
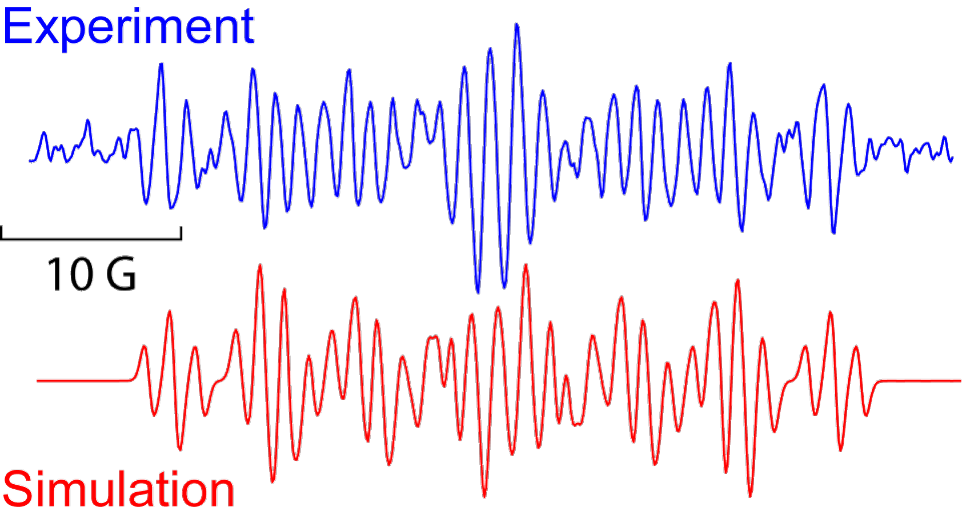
EPR spectrum during photolysis of **2a** in *t*-BuPh at 230 K. Top (blue): experiment; bottom (red): simulation.

EPR spectra from oxime carbonate **2a**, containing a benzofuran acceptor, gave well resolved spectra only at 230–235 K (Figure 3). The corresponding benzofuranyl-iminyl was not detectable at 230 K or above.

**Scheme 2:**
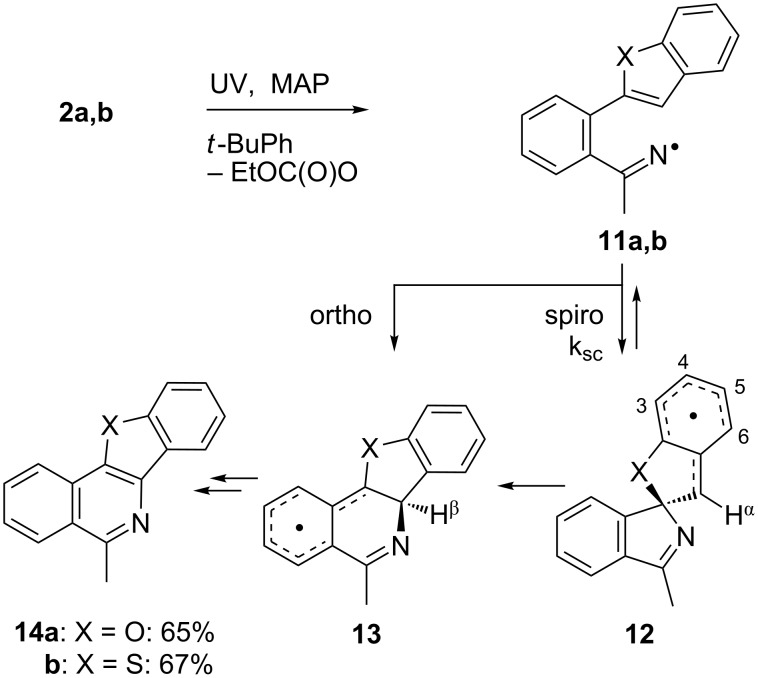
Ring closure of iminyl radicals derived from **2a**,**b**.

The EPR hfs obtained from simulation of the spectrum (Table 2) show this to be a benzyl type radical and we assign it structure **12a** (Scheme 2). A DFT computation for **12a** at the UB3LYP/6-311+G(2d,p) level of theory gave hfs in close agreement with experiment (Table 2). The EPR hfs are certainly not consistent with structure **13**, which was not spectroscopically detected. It is evident therefore that iminyl radical **11a** rapidly and selectively undergoes spiro-cyclisation with the benzofuran acceptor.

**Table 2:** EPR parameters of spiro*-*benzyl radicals **12** derived from **2a,b**^a^.

Radical	T/K or DFT method	*g*-factor	*a*(H^α^)	*a*(H^3^)	*a*(H^4^)	*a*(H^5^)	*a*(H^6^)	*a*(N)

**12a** (X = O)	230	2.0025	14.9	1.4	6.3	1.4	5.0	5.4
**12a** (X = O)	UB3LYP/6-311+G(2d,p)	–	−14.2	1.6	−5.9	1.6	−5.0	5.2
**12b** (X = S)	230	–	13.0	1.5	6.0	1.5	5.3	4.1
**12b** (X = S)	UB3LYP/6-311+G(2d,p)		−13.9	2.0	−5.9	2.0	−5.1	5.8

^a^At 9.4 GHz in *t*-BuPh solution; hfs in Gauss.

Rate constants of ring-closure reactions can be determined for sterically unhindered radicals by measurements of the concentrations of the ring-open and cyclised radicals under EPR conditions [43–45]. Steady-state concentrations of **12a** were determined in the usual way from the spectra [46].

We estimated that the concentration ratio [**12a**]/[**11a**] > 4 at 230 K, and hence *k*_sc_(230 K) > 55 s^−1^ (see Supporting Information File 1 for details). Most radical cyclisations have Arrhenius log(*A*_c_) ≈ 10.5 s^−1^ [47–48] and, by assuming that this holds for **12a**, an activation energy *E*_sc_ < 9 kcal mol^−1^ and *k*_sc_(300 K) > 5 × 10^3^ s^−1^ are obtained (see Supporting Information File 1). The rate constant for *5-exo*-cyclisation of the phenylpentenyliminyl radical **15** was reported [34] to be *k*_c_(300 K) = 8.8 × 10^3^ s^−1^ with *E*_c_ = 8.3 kcal mol^−1^ and therefore, particularly in view of the large resonance stabilisation of spiro*-*cyclised radical **12a**, these rate parameters seem to be very reasonable estimates (Table 3).

**Table 3:** Rate data for spiro- and other cyclisations of C- and N-centred radicals.

Entry	Radical	Structure	Mode	log(*A*_c_/s^−1^)^a^	*E*_c_/kcal mol^−1^	*k*_c_(300 K)/s^−1^	Ref.

1	**16**	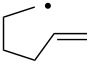	*5-exo*	10.4	6.85	2.3 × 10^5^	[49]
2	**15**	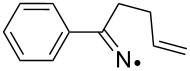	*5-exo*	[10.0]	8.3	8.3 × 10^3^	[34]
3		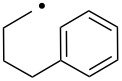	spiro			<5 × 10^4^ (323 K)	[50]
4		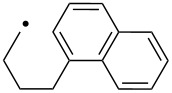	spiro			<10^4^ (353 K)	[51]
5	**5b**	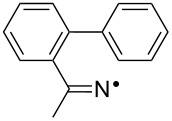	ortho			<5 × 10^3^	this work
6	**12a**	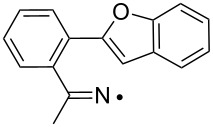	spiro	[10.5]	< 9	>5 × 10^3^	this work

^a^Values in parenthesis assumed.

Curiously, analysis of the products from a photolysis of **2a** carried out at higher temperature (rt) in benzotrifluoride solvent, showed benzofuro[3,2-*c*]isoquinoline derivative **14a** to be the main product (65%) [26]. This implied ortho-radicals **13** as intermediates and appeared to conflict with the EPR result. The most likely explanation is that, at the temperature of the preparative experiments (~100 K higher than the EPR study) the spiro-cyclisation is reversible whereas the 6-ortho-process is not. The ortho-product then accumulates because of thermodynamic control. Alternatively, spiro-radical **12a** might rearrange via a tetracyclic aziridinyl intermediate (or transition state) at higher temperatures. DFT computations (see below) undermined this possibility however.

EPR experiments with the benzothiophene-containing precursor **2b** showed a complex spectrum from at least two radicals. A reasonable simulation (see Supporting Information File 1) was obtained as a combination of the spiro-radical **12b** (hfs in Table 2) and the solvent-derived adduct **9a**. Most likely therefore spiro-cyclisation predominates at low temperature but again thermodynamic control takes over at higher temperatures because **14b** was isolated as the main product.

EPR spectra from the furan-containing precursor **3**, and from the more flexible aromatic precursor **4**, revealed only the corresponding ring-open iminyl radicals and neither spiro- nor ortho-radicals in the temperature range 230–260 K. It can be concluded that the rates of their iminyl ring closures are significantly slower. This accords with expectation, because the cyclised radical from **3**, without the benzo-ring of **12**, would be less thermodynamically stabilised. The iminyl radical from **4** has a more flexible chain and this probably accounts for its slower ring closure.

Kinetic data for iminyl radical ring closures is compared with analogous data for model C*-*centred radicals in Table 3. As mentioned above, biphenyl-iminyl radicals **5** were spectroscopically detectable at 270 K; well above the temperature at which **12a** underwent spiro-cyclisation. It follows that *k*_c_(300 K) for **5b** must be < *k*_c_ for **12a** and this information is included in Table 3.

The rate constant for *5-exo*-cyclisation the archetype iminyl **15** (Table 3, entry 2) is more than an order of magnitude smaller than the rate constant for hex-5-enyl (**16**, Table 3, entry 1) [49]. Our rates for ortho*-* (Table 3, entry 5) and spiro*-* (Table 3, entry 6) cyclisations of iminyls onto aromatics were also slower than for C-centred radicals (Table 3, entries 3 and 4) [50–51]. The pattern of slower cyclisations for iminyls compared with alkyls seems established for both alkene- and arene-type acceptors.

### QM Computations

To shed further light on the spiro versus ortho alternatives we computed the activation parameters [Δ*E*^‡^_298_] and reaction enthalpies [Δ*H*_298_], corrected to 298 K for thermal effects, for the spiro- and ortho-cyclisation modes of a representative set of aryliminyl radicals (Table 4). It was known that the DFT UB3LYP/6-31+G(d) and UB3LYP/6-311+G(2d,p) methods gave results in reasonable harmony with experiment for related iminyl radicals [34] and therefore these methods were adopted. For each process the two basis sets gave results in reasonable agreement (Table 4). The cyclisations of the flexible iminyl radicals **4Im** (Tabel 4, entries 1 and 2) were predicted to have the highest activation energies in either mode and to be strongly endothermic. This accords well with the absence of cyclised species in the EPR spectra and with the lack of cyclised products.

**Table 4:** DFT computed activation energies (Δ*E^‡^*_298_) and reaction enthalpies (Δ*H*_298_) in kcal mol^−1^ for aromatic iminyl radicals.

Entry	Iminyl	Product	Method^a^	Δ*E^‡^*_298_	Δ*H*_298_

1	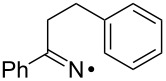 **4Im**	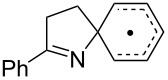 **4sp**	A B	16.6 16.9	6.6 7.3
2	**4Im**	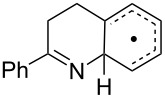 **4or**	B	19.9	8.0
3	**4Im**	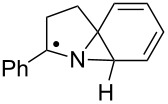 **4az**	B		32.9
4	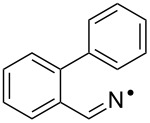 **5a**	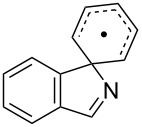 **7a**	A B	14.7 15.0	7.0 7.6
5	**5a**	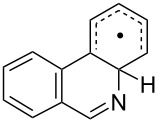 **8a**	A B	11.3 11.6	−2.8 −2.1
6	**5a**	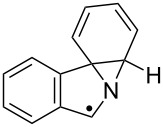 **5az**	B		29.5
7	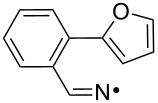 **3Im**	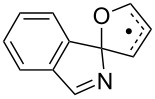 **3sp**	A B	11.6 12.1	−1.1 −0.5
8	**3Im**	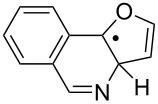 **3en**	A B	14.2 14.7	−2.3 −1.8
9	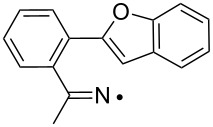 **11a**	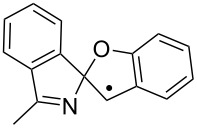 **12a**	A B	8.9 9.3	−7.8 −6.9
10	**11a**	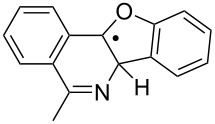 **13a**	A B	12.6 13.1	−8.6 −8.1
11	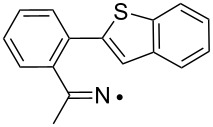 **11b**	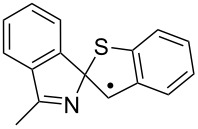 **12b**	B	9.5	−6.2
12	**11b**	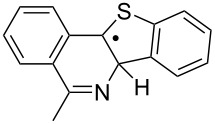 **13b**	B	10.5	−11.3

**^a^**A = UB3LYP/6-31+G(d), B = UB3LYP/6-311+G(2d,p).

For the parent biphenyliminyl radical **5a** the ortho*-*ring closure (Table 4, entry 5) was computed to have a lower activation energy than spiro-cyclisation (Table 4, entry 4) and to be exothermic in comparison with the endothermic spiro-mode. This was in good accordance with the observed exclusive formation of phenanthridines derived from radicals **5a–f** for these compounds. The possibility of the rearrangement of spiro*-* to ortho*-*species via aziridinyl intermediates (or transition states) (**az**) which contain benzyl stabilization was examined. However, the computations for **4az** (Table 4, entry 3) and **5az** (Table 4, entry 6) found these processes to be strongly endothermic, so they can probably be ruled out of consideration. Predicted activation energies and reaction enthalpies for spiro- (Table 4, entry 7) and ortho-ring closure (Table 4, entry 8) of the furanyl-iminyl **3Im**, suggested spiro*-*closure would be favoured. However, the magnitude of Δ*E^‡^*_298_ is close to that for ortho*-*closure of the biphenyl analogue **5a**. Thus cyclised products might be obtainable from photolyses of **3** at higher temperatures.

For the benzofuranyl- and benzothiophenyl-iminyl radicals **11a** and **11b** (Table 4, entries 9–12) the computations predicted a reversal in the preferred mode of ring closure: Δ*E^‡^*_298_ values for spiro-cyclisation were significantly lower than for ortho-cyclisation and lower than for **5a.** This agreed well with the EPR detection of the spiro-intermediates **12a** and **12b**. From the EPR experiments the *E*_sc_ of < 9 kcal mol^−1^ for spiro-cyclisation of **11a** (see above Table 3) was close to the computed Δ*E^‡^*_298_ values of 8.9 and 9.3 kcal mol^−1^ (Table 4, entry 9). For both **11a** and **11b**, although the computed Δ*E^‡^*_298_ values for ortho-ring closure are significantly higher than for spiro-ring closure, the ortho-processes are computed to be more exothermic. Thus, theory supports the idea, derived from the isolation of products from ortho-closure of **14a**,**b**, that thermodynamic control supervenes at higher temperatures.

**Figure 4:**
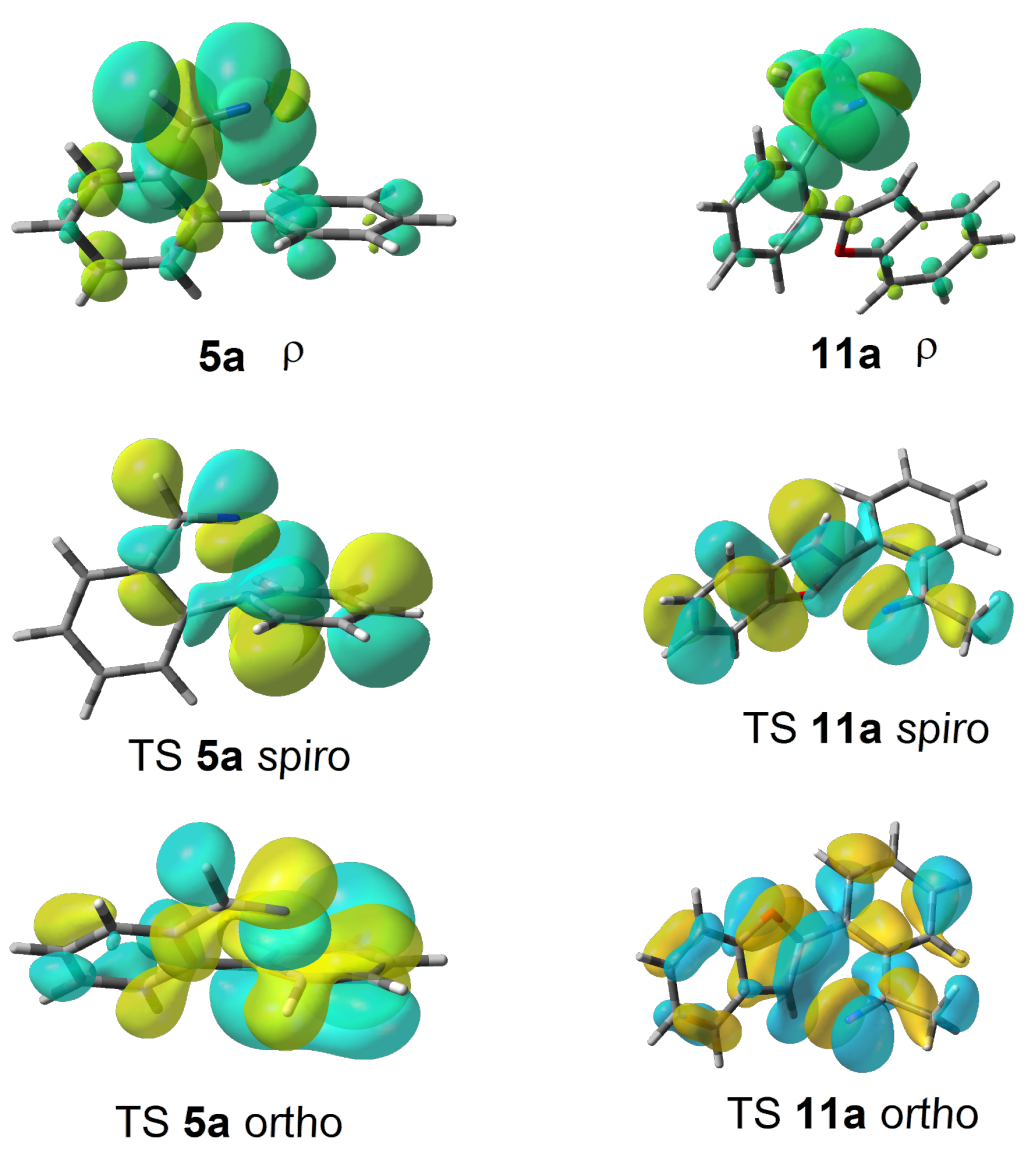
DFT computed structures for **5a**, **11a** and their cyclisation transition states (TS). Top line: spin density distribution maps for iminyls **5a** and **11a**. Middle line: spiro TSs, r(N···C) for **5a** = 1.928 and for **11a** = 2.064 Å. Bottom line: ortho TSs, r(N···C) for **5a** = 1.954 and for **11a** = 2.027 Å.

Some clues as to why the biphenyl-iminyls **5** prefer the ortho mode whereas the benzofuranyl- and benzothiophenyl-iminyls **11** prefer the spiro mode can be obtained by consideration of the structures of the transition states (TS, Figure 4). The top line shows the computed spin density (ρ) maps for the reactant iminyls. In agreement with the EPR spectra, the spin density is largely concentrated in orbitals in the plane of the iminyl units. The middle line of Figure 4 shows the TS structures and SOMOs for spiro-cyclisations of **5a** and **11a**. For both radicals the iminyl units approach the acceptor rings from vertically above; the angle between the ArC(R)=N unit and the plane of the acceptor ring is ~90° for both TSs. This ensures that overlap between the incoming iminyl orbital and the acceptor ring orbitals is optimum. The bottom line shows the TS structures and SOMOs for the ortho-cyclisations of **5a** and **11a**. For both radicals the architectures oblige the iminyl units to approach the acceptor rings at much more oblique angles. Thus, overlap of the in-plane iminyl orbital with the acceptor ring orbitals is poorer than for spiro-cyclisation. However, this is offset by the greater resonance delocalisation in the TS frontier orbitals for ortho-cyclisation (see Figure 4). There is a trade-off between these two factors and the default is that the resonance delocalisation factor in ortho-cyclisation prevails. Evidently **11a**,**b**, are exceptional in that the iminyl/acceptor overlap factor outweighs the resonance delocalisation factor.

## Conclusion

Ethoxycarbonyl oximes of aromatic and heteroaromatic ketones are easily made and handled and have long shelf lives. We have found them to be outstanding precursors for the photochemical generation of aromatic iminyl radicals. Product analyses, EPR spectroscopic observations and DFT computations all converged in indicating that the “default” ring closure mode for iminyl radicals is ortho-cyclisation onto suitably situated aromatic rings. The exception to this rule was spiro-cyclisation onto benzofuran and benzothiophene rings at low temperatures. Even for these acceptors, however, thermodynamic control ensured that the only isolable products were from ortho-closures. Rate parameters for the spiro-cyclisation of **11a**, estimated by the steady-state kinetic EPR method, showed the process to be about an order of magnitude slower than for archetype C-centred radicals. This was in harmony with the generally slower reaction rates of iminyl radicals compared with alkyl radicals. DFT-computed energetics were in good agreement with experiment and supported the idea of thermodynamic control for the cyclisation of **11a**,**b**.

## Experimental

EPR spectra were obtained at X-Band on Bruker EMX 10/12 spectrometers at St. Andrews and Manchester. Oxime carbonates (2 to 15 mg) and MAP (1 equiv wt/wt) in *t*-BuPh or benzene (0.5 mL) were prepared in quartz tubes and deaerated by bubbling with nitrogen for 15 min. Photolysis in the resonant cavity was by unfiltered light from a 500 W super-pressure mercury arc lamp or, in the Manchester experiments, from a Luxtel CL300BUV lamp. EPR signals were digitally filtered and double integrated by using the Bruker WinEPR software, and radical concentrations were calculated by reference to the double integral of the signal from a known concentration of the stable radical DPPH run under identical conditions. The majority of EPR spectra were recorded with 2.0 mW power, 0.8 G_pp_ modulation intensity and gain of 10^6^.

QM calculations were carried out by using the Gaussian 09 program package. Geometries were fully optimised for all model compounds. Optimised structures were characterised as minima or saddle points by frequency calculations. The experimental kinetic and spectroscopic data was all obtained in the nonpolar hydrocarbon solvents *t-*BuPh or cyclopropane. Solvent effects, particularly differences in solvation between the neutral reactants and neutral transition states, were therefore expected to be minimal. In view of this, no attempt was made to model the effect of the solvent computationally.

## Supporting Information

File 1 General procedures. Preparation and characterization data of oxime carbonates. Sample EPR spectra and kinetic data. ^1^H and ^13^C NMR spectra for novel compounds.
