# Supplementary material for: Interplay of ortho- with spiro-cyclisation during iminyl radical closures onto arenes and heteroarenes
Source: Beilstein J Org Chem. 2013 Jun 4;9:1083–92. doi: 10.3762/bjoc.9.120 (PMC3678706; doi:10.3762/bjoc.9.120)
Supplement: File 1 — General procedures. Preparation and characterization data of oxime carbonates. Sample EPR spectra and kinetic data. 1H and 13C NMR spectra for novel compounds. [file Beilstein_J_Org_Chem-09-1083-s001.pdf]

Supporting Information

for

Interplay of ortho- with spiro-cyclisation during iminyl radical closures onto arenes and heteroarenes

Roy T. McBurney* and John C. Walton*

Address: EaStCHEM School of Chemistry, University of St. Andrews, St. Andrews, Fife, KY16 9ST, UK

Email: Roy T. McBurney - roy.mcburney@glasgow.ac.uk; John C. Walton - jcw@st-and.ac.uk

* Corresponding author

General procedures. Preparation and characterization data of oxime carbonates. Sample EPR spectra and kinetic data. ^{1}H and ^{13}C NMR spectra for novel compounds

Table of Contents

General experimental section S3

Synthesis and experimental section S3

EPR spectroscopy S7

EPR spectra S9

Computational methods S12

References S12

^{1}H and ^{13}C NMR spectra of novel compounds S14

General experimental section

All reagents and solvents were purchased from either Sigma Aldrich or Alfa Aesar and used without further purification. Toluene and tetrahydrofuran were distilled over sodium, and dichloromethane was distilled over calcium hydride. Benzaldehyde oxime and acetophenone oxime were prepared according to the literature procedure [1], as was *N*-benzylpent-4-en-1-amine [2]. Column chromatography was carried out using Silica 60A (particle size 40–63 µm, Silicycle, Canada) as the stationary phase, and TLC was performed on precoated silica gel plates (0.20 mm thick, Sil G UV_{254}, Macherey-Nagel, Germany) and observed under UV light. ^{1}H and ^{13}C NMR spectra were recorded on Bruker AV III 500, Bruker AV II 400 and Bruker AV 300 instruments. Chemical shifts are reported in parts per million (ppm) from low to high frequency and referenced to the residual solvent resonance. Coupling constants (*J*) are reported in hertz (Hz). Standard abbreviations indicating multiplicity were used as follows: s = singlet, d = doublet, t = triplet, dd = double doublet, q = quartet, m = multiplet, b = broad. Melting points (mp) were determined using a Sanyo Gallenkamp apparatus and are reported uncorrected. Mass spectrometry was carried out at the EPSRC National Mass Spectrometry Service Centre, Swansea, UK.

Synthesis and experimental section

Oxime carbonates **1a–f**, and **2a**,**b** were prepared as described previously [1].

1-(2-(Furan-2-yl)phenyl)ethan-1-one

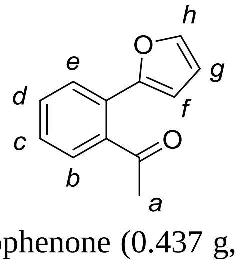

To a stirred solution of 2-bromoacetophenone (0.437 g, 2.19 mmol, 1.0 equiv) and 2-furanyl boronic acid (0.295 g, 2.64 mmol, 1.2 equiv) in a toluene/ethanol (4:1, 60 mL) mixture was

added potassium carbonate (0.911 g, 6.60 mmol, 3.0 equiv) and tetrakis(triphenylphosphine) palladium(II) (0.254 g, 0.22 mmol, 0.1 equiv). The resulting suspension was heated under reflux in an atmosphere of Ar for 18 h. The solvent was removed under reduced pressure, and the crude residue was redissolved in H_2O (100 mL) and extracted with EtOAc (3 × 100 mL). The combined organic layers were washed with brine (100 mL) and dried over $MgSO_4$. The filtrate was concentrated under reduced pressure and purified by column chromatography (1:1 CH_2Cl_2/petrol ether 40:60 to CH_2Cl_2, gradient elution) to yield the title compound as a colourless oil (0.409 g, yield 99%). ^{1}H NMR (400 MHz, $CDCl_3$, 296 K) δ 2.26 (s, 3H, H_a), 6.50 (dd, *J* = 3.3 Hz, 1.8 Hz, 1H, H_g), 6.59 (dd, *J* = 3.3 Hz, 0.7 Hz, 1H, H_f), 7.36 (m, 1H, H_c), 7.42 (d, *J* = 8.0 Hz, 1H, H_e), 7.47 (m, 1H, H_d), 7.51 (dd, *J* = 1.8 Hz, 0.7 Hz, 1H, H_h), 7.58 (d, *J* = 8.69 Hz, 1H, H_b); ^{13}C NMR (100 MHz, $CDCl_3$, 296 K) δ 29.8, 108.4, 112.0, 127.2, 127.7, 128.0, 128.3, 130.4, 139.6, 143.1, 152.3, 204.7.

1-(2-(Furan-2-yl)phenyl)ethan-1-one oxime

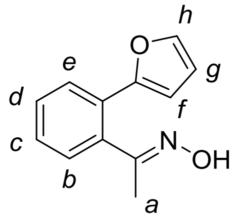

To a stirred solution of 1-(2-(furan-2-yl)phenyl)ethan-1-one (0.285 g, 1.66 mmol, 1.0 equiv) in EtOH (20 mL) was added hydroxylamine hydrochloride (0.230 g, 3.31 mmol, 2.0 equiv) and sodium acetate (0.265 g, 3.31 mmol, 2.0 equiv). The resulting suspension was heated under reflux for 18 h. The solvent was removed under reduced pressure and the crude residue was redissolved in H_2O (100 mL) and extracted with CH_2Cl_2 (3 × 100 mL). The combined organic layers were dried over $MgSO_4$, concentrated under reduced pressure and used without further purification (0.328 g). ^{1}H NMR (400 MHz, $CDCl_3$, 296 K) δ 1.92 (s, 3H, H_a), 6.37–6.39 (m, 1H, H_g), 6.43 (d, *J* = 3.0 Hz, 1H, H_f), 7.21–7.36 (m, 3H, $H_{c,d,e}$), 7.42 (d, *J* = 1.1

Hz, 1H, H*h*), 7.59 (d, *J* = 7.9 Hz, 1H, H*b*), 8.90 (br, 1H, H*OH*); ^{13}C NMR (100 MHz, $CDCl_3$, 296 K) δ 15.6, 108.8, 111.7, 127.4, 127.7, 128.9, 129.3, 129.4, 135.0, 142.6, 152.3, 159.0.

1-(2-(Furan-2-yl)phenyl)ethanone *O*-ethoxycarbonyl oxime (3)

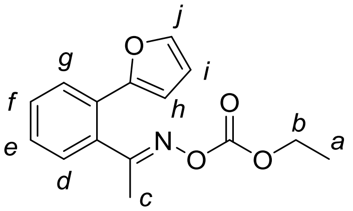

To a 0 °C solution of the oxime (0.36 g, 1.76 mmol, 1.0 equiv) and pyridine (0.14 mL, 1.0 equiv) in CH_2Cl_2 (20 mL) was added dropwise ethyl chloroformate (0.17 mL, 1.0 equiv). The yellow solution was allowed to warm to rt and stirred for 18 h. The reaction mixture was diluted with CH_2Cl_2 (100 mL) and washed with 1 M HCl (100 mL), sat. aqueous $NaHCO_3$ (100 mL) and brine (100 mL), dried ($MgSO_4$), concentrated under reduced pressure, and purified by column chromatography (1:1 CH_2Cl_2/petrol ether) to yield the title compound as a colourless oil, 0.429 g, yield 89%. ^{1}H NMR (400 MHz, $CDCl_3$, 296 K) δ 1.24 (t, *J* = 7.1 Hz, 3H, H*a*), 2.24 (s, 3H, H_c), 4.19 (q, *J* = 7.1 Hz, 2H H_b), 6.44–6.5 (m, 1H, H_i), 6.60, (d, *J* = 3.4 Hz, 1H, H_h), 7.13 (d, *J* = 7.7 Hz, 1H, H*d*), 7.33 (td, *J* =1.3, 7.5 Hz, 1H, H*Ar*), 7.41 (td, *J* = 1.3, 7.5 Hz, 1H, H*Ar*) 7.47 (d, *J* = 1.7 Hz, 1H, H_{Ar}), 7.73 (d, *J* = 7.9 Hz, 1H, H_{Ar}); ^{13}C NMR (100 MHz, $CDCl_3$, 294 K) δ 14.4, 17.8, 64.7, 109.1, 111.9, 127.4, 127.8, 129.3, 129.7 (x2), 133.0, 142.9, 151.9, 153.9, 166.4.

1,3-Diphenylpropan-1-one oxime

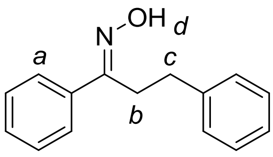

A suspension of 1,3-diphenylpropan-1-one (3.045 g, 14.5 mmol, 1.0 equiv), hydroxylamine hydrochloride (2.012 g, 28.9 mmol, 2.0 equiv) and sodium acetate (2.371 g, 28.9 mmol, 2.0 equiv) in EtOH (100 mL) was heated under reflux for 18 h. The solvent was removed under reduced pressure and the crude residue redissolved in H_2O (100 mL) and extracted into

CH_2Cl_2 (3 × 100 mL). The combined organic layers were dried ($MgSO_4$) and concentrated under reduced pressure to yield the title compound as a colourless solid, 3.088 g, yield 94%. Mp 77 °C: ^{1}H NMR (400 MHz, $CDCl_3$, 294 K) δ 2.89–2.94 (m, 2H, H_c), 3.11–3.15 (m, 2H, H_b), 7.19–7.32 (m, 5H, H_{Ar}), 7.40–7.43 (m, 3H, H_{Ar}), 7.59–7.61 (m, 2H, H_a), 8.02–8.49 (br, 1H, H_d); ^{13}C NMR (100 MHz, $CDCl_3$, 294 K) δ 29.0, 32.1, 126.3, 126.6, 128.4, 128.5, 128.7, 129.7, 134.9, 141.2, 159.6; ESIMS *m/z*: 226 $[MH]^+$; HR-ESIMS *m/z*: $[MH]^+$ calcd. for $C_{15}H_{16}NO$, 226.1226; found, 226.1228.

1,3-Diphenylpropan-1-one *O*-ethoxycarbonyl oxime (4)

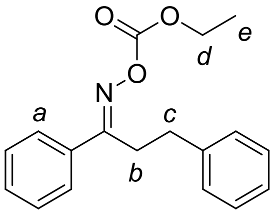

To a 0 °C solution of 1,3-diphenylpropan-1-one oxime (1.064 g, 4.72 mmol, 1.0 equiv) and pyridine (0.83 mL, 10.38 mmol, 2.2 equiv) in CH_2Cl_2 (40 mL) was added dropwise ethyl chloroformate (0.90 mL, 9.44 mmol, 2.0 equiv), and the yellow solution was allowed to warm to rt and stirred for 18 h. The reaction mixture was diluted with CH_2Cl_2 (100 mL) and washed with 1 M HCl (100 mL), sat. aqueous $NaHCO_3$ (100 mL), and brine (100 mL), dried ($MgSO_4$), concentrated under reduced pressure, and purified by column chromatography (1:1 CH_2Cl_2/Petrol Ether) to yield the title compound as a colourless oil, 1.094 g, yield 80%. ^{1}H NMR (400 MHz, $CDCl_3$, 294 K) δ 1.40 (t, J = 7.1 Hz, 3H, H_e), 2.88–2.92 (m, 2H H, H_b), 3.14–3.18 (m, 2H, H_c), 4.35 (q, J = 7.1 Hz, 2H, H_d), 7.20–7.32 (m, 5H, H_{Ar}), 7.39–7.48 (m, 3H, H_{Ar}), 7.70 (dd, J = 1.3 Hz, 8.0 Hz, 2H, H_a); ^{13}C NMR (100 MHz, $CDCl_3$, 294 K) δ 14.4, 30.2, 32.6, 64.7, 126.4, 127.3, 128.3, 128.6, 128.7, 130.6, 133.7, 140.4, 153.9, 165.4; ESIMS *m/z*: 298 $[MH]^+$; HR-ESIMS *m/z*: $[MH]^+$ calcd. for $C_{18}H_{20}NO_3$, 298.1438; found, 298.1442.

UV cyclisation of oxime carbonate derivatives general procedure

A quartz tube was charged with oxime carbonate (1.0 equiv), 4-methoxyacetophenone (MAP) (1 equiv wt/wt) and benzotrifluoride (3 mL). The reaction mixture was degassed by bubbling Ar through the solution for 15 min. The solution was irradiated with UV light (400 W medium pressure Hg lamp) for 3 h. The solvent was removed under reduced pressure and the crude residue purified by column chromatography (CH_2Cl_2/EtOAc 9:1 as eluent).

UV photolysis of 1,3-diphenylpropan-1-one *O*-ethoxycarbonyl oxime (4)

According to the general procedure with compound (**4**) (56 mg) and MAP (56 mg) in $PhCF_3$ (2.0 mL). The total product mixture, after solvent removal was examined by ^1H NMR and GC-MS. t_R/min; 13.6 (M^+ 210, 1,3-diphenylpropan-1-one), 13.9 (M^+ 209, 1,3-diphenylpropan-1-imine), 25.3 (M^+ > 397, probably Im_2). The main fraction after chromatography was shown by ^1H NMR to be a mixture of 1,3-diphenylpropan-1-one and 1,3-diphenylpropan-1-imine together with several unidentified components. The product of ortho-cyclisation, 2-phenyl-3,4-dihydroquinoline, is a known compound [3] and was shown not to be present.

EPR spectroscopy

EPR spectra were obtained at 9.5 GHz with 100 kHz modulation employing a Bruker EMX 10/12 spectrometer fitted with a rectangular ER4122 SP resonant cavity and a Bruker ER4122-SHQE X band cavity on EMX and EMX Micro consoles in Manchester. Stock solutions of each oxime carbonate (2 to 15 mg) and MAP (1 equiv wt/wt) in *tert*-butylbenzene or benzene (0.5 mL) were prepared and sonicated where necessary. An aliquot (0.2 mL), to which any additional reactant had been added, was placed in a 4 mm o.d. quartz tube and deaerated by bubbling nitrogen for 15 min. Photolysis in the resonant cavity was by

unfiltered light from a 500 W super pressure mercury arc lamp or, in the Manchester experiments, the light source was a Luxtel CL300BUV lamp. Solutions in cyclopropane were prepared on a vacuum line by distilling in the cyclopropane, degassing with three freeze-pump-thaw cycles and finally flame sealing the tubes. In all cases where spectra were obtained, hfs were assigned with the aid of computer simulations using the Bruker SimFonia and NIEHS Winsim2002 software packages. For kinetic measurements, precursor samples were used mainly in "single shot" experiments, i.e., new samples were prepared for each temperature and each concentration to minimise sample-depletion effects. EPR signals were digitally filtered and double integrated by using the Bruker WinEPR software and radical concentrations were calculated by reference to the double integral of the signal from a known concentration of the stable radical DPPH [1×10^{-3} M in PhMe], run under identical conditions, as described previously. The majority of EPR spectra were recorded with 2.0 mW power, 0.8 G_{pp} modulation intensity, and a gain of ca. 10^6.

Sample EPR spectra from biphenyl-based oxime carbonates 1a–f and 4.

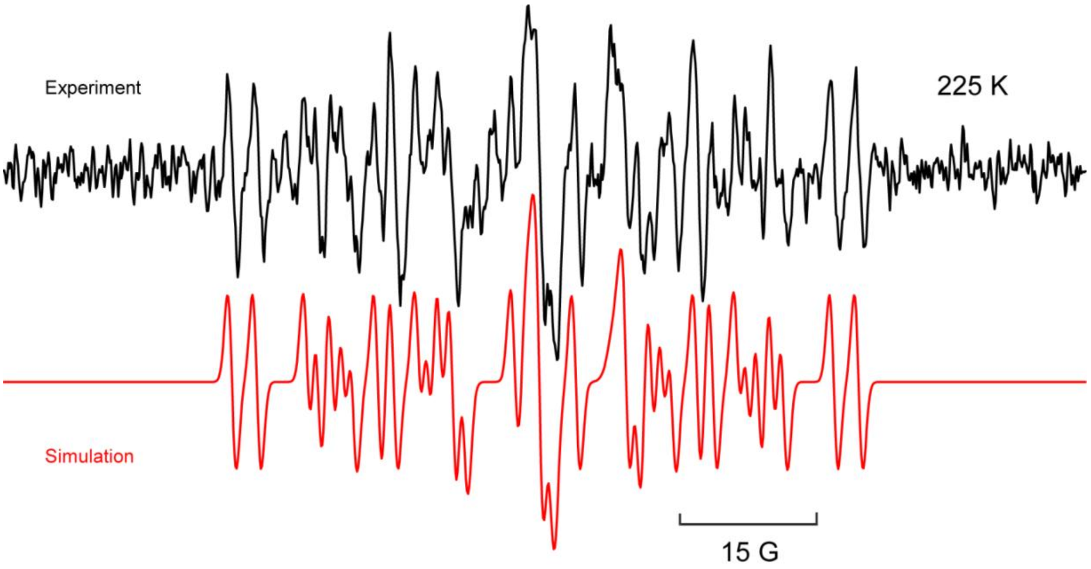

Figure S1: EPR spectrum obtained during UV photolysis of 1-(4'-(trifluoromethyl)-[1,1'-biphenyl]-2-yl)ethanone *O*-ethoxycarbonyl oxime (**1d**) in *t*-BuPh at 225 K.

Top: experiment; bottom: computer simulation.

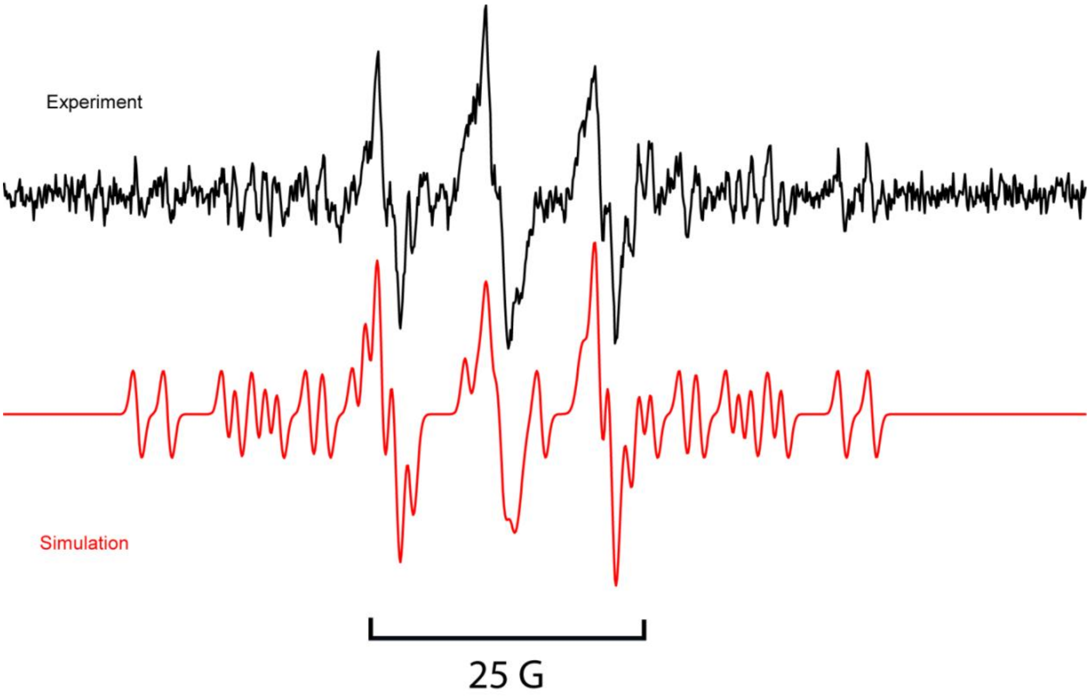

Figure S2: EPR spectrum obtained during UV photolysis 1-(4'-methyl-[1,1'-biphenyl]-2-yl)ethanone O-ethoxycarbonyl oxime (**1b**) in *t*-BuPh at 225 K.

Top: experiment; bottom: computer simulation.

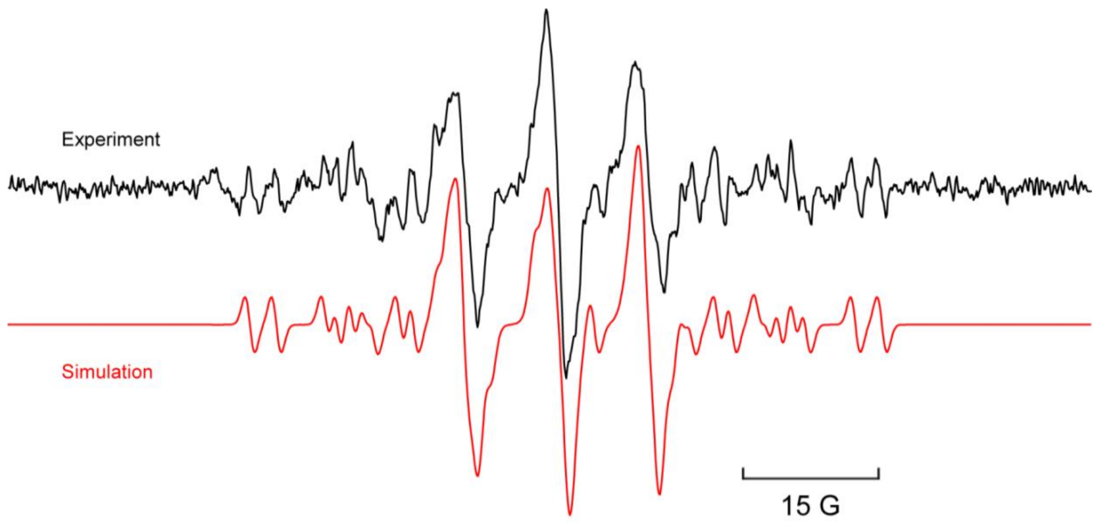

Figure S3: EPR spectrum obtained during UV photolysis of 1,3-diphenylpropan-1-one *O*-ethoxycarbonyl oxime (**4**) in *t*-BuPh at 225 K. Top: experiment; bottom: computer simulation.

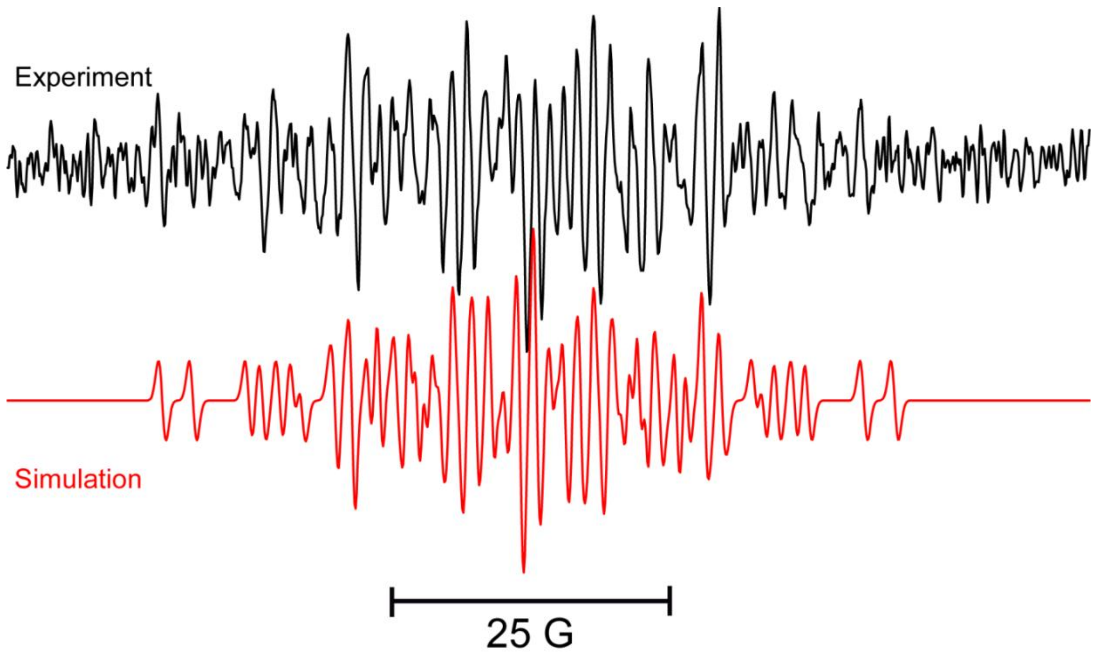

Figure S4: EPR spectrum obtained during UV photolysis 1-(2-(benzo[*b*]thiophen-2-yl)phenyl)ethanone O-ethoxycarbonyl oxime (**2b**) in *t*-BuPh at 230 K. Top: experiment; bottom: computer simulation.

Table S1: Estimate of spirocyclization rate data from kinetic steady state EPR of benzofurano-iminyl radical (**11a**), UV in *t*-butylbenzene + MAP

Dial Temp.	Actual T/K	scans		Gain	[Sp]/[Im]	D. Int. Spiro	[Im] M	[Spiro] M	Im-Spiro M	$k_{sp}/2kt$	$\log k_{sp}/2kt$	log2kt n-C7	$10^3/T$	log η n-C7	log η t-BuPh	log2kt t-BuPh	$\log k_{sp}$ t-BuPh	k_{sp} s-1
230	230.93	20		4.0E+06	4	39.7	4.0E-09	1.0E−08	1.20E−08	8.01E−08	−7.097	9.498	4.330	0.00	0.66	8.834	1.74E+00	5.46E+01
													0.000				1.05E+01	

[DPPH]	1.0E-03
Gain DPPH	2.0E+03
Doub. Int DPPH	49
temp DPPH	290
Actual T	292.1
F[cyc]	1
F[Im]	1

Computational methods

Radical ground-state calculations were carried out by using the Gaussian 09 program package [4]. Becke's three-parameter hybrid exchange potential (B3) was used with the LYP correlation functional, B3LYP. This method has previously described the chemistry of iminyl radicals accurately. The standard split-valence 6-31+G(d) basis set was initially employed and then the computations were extended to the UB3LYP/6-311+D(2d,p) level. Geometries were fully optimised for all model compounds. Optimised structures were characterised as minima or saddle points by frequency calculations. The experimental kinetic and spectroscopic data was all obtained in the nonpolar hydrocarbon solvents *tert*-butylbenzene or cyclopropane. Solvent effects, particularly differences in solvation between the neutral reactants and neutral transition states, are therefore expected to be minimal. In view of this, no attempt was made to computationally model the effect of the solvent.

References

[1] (a) McBurney, R. T.; Slawin, A. M. Z.; Smart, L. A.; Yu, Y.; Walton, J. C. *Chem. Commun*. **2011**, *47*, 7974–7976; (b) McBurney, R. T.; Harper, A. D.; Slawin, A. M. Z. Walton, J. C. *Chem. Sci*. **2012**, *3*, 3436–3444.

[2] Gribkov, D. V.; Hultzsch, K. C. *Angew. Chem. Int. Ed.*, **2004**, *43*, 5542.

[3] Wang, T.; Zhuo, L.-G.; Li, Z.; Chen F.; Ding Z.; Y.; Fan, Q.-H.; Xiang, J.; Yu, Z.; Chan, A. S. C. *J. Am. Chem. Soc.*, **2011**, *133*, 9878–9891.

[4] Gaussian 09, Revision A.02, M. J. Frisch, G. W. Trucks, H. B. Schlegel, G. E. Scuseria, M. A. Robb, J. R. Cheeseman, G. Scalmani, V. Barone, B. Mennucci, G. A. Petersson, H. Nakatsuji, M. Caricato, X. Li, H. P. Hratchian, A. F. Izmaylov, J. Bloino, G. Zheng, J. L. Sonnenberg, M. Hada, M. Ehara, K. Toyota, R. Fukuda, J. Hasegawa, M. Ishida, T. Nakajima, Y. Honda, O. Kitao, H. Nakai, T. Vreven, J. A. Montgomery, Jr., J. E. Peralta, F. Ogliaro, M. Bearpark, J. J. Heyd, E. Brothers, K. N. Kudin, V. N. Staroverov, R.

Kobayashi, J. Normand, K. Raghavachari, A. Rendell, J. C. Burant, S. S. Iyengar, J. Tomasi, M. Cossi, N. Rega, J. M. Millam, M. Klene, J. E. Knox, J. B. Cross, V. Bakken, C. Adamo, J. Jaramillo, R. Gomperts, R. E. Stratmann, O. Yazyev, A. J. Austin, R. Cammi, C. Pomelli, J. W. Ochterski, R. L. Martin, K. Morokuma, V. G. Zakrzewski, G. A. Voth, P. Salvador, J. J. Dannenberg, S. Dapprich, A. D. Daniels, O. Farkas, J. B. Foresman, J. V. Ortiz, J. Cioslowski, and D. J. Fox, Gaussian, Inc., Wallingford CT, 2009.

^{1}H and ^{13}C NMR spectra of novel compounds

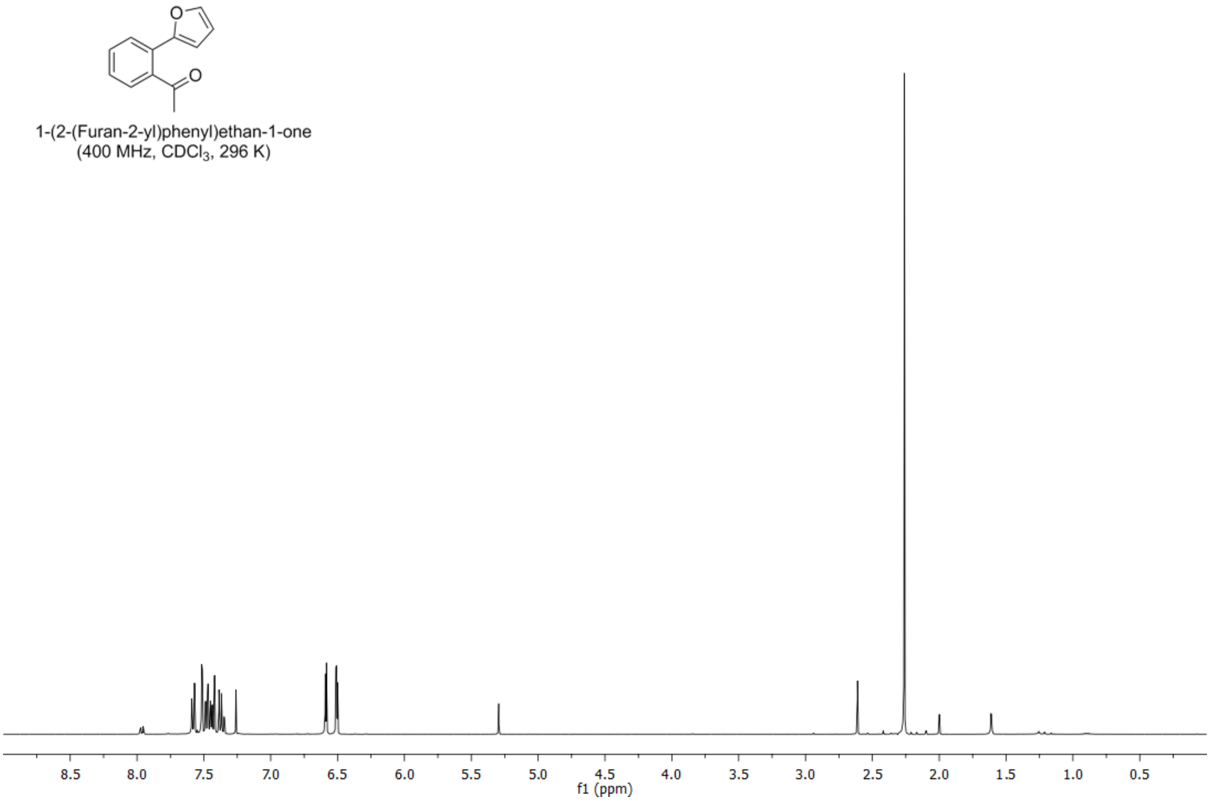

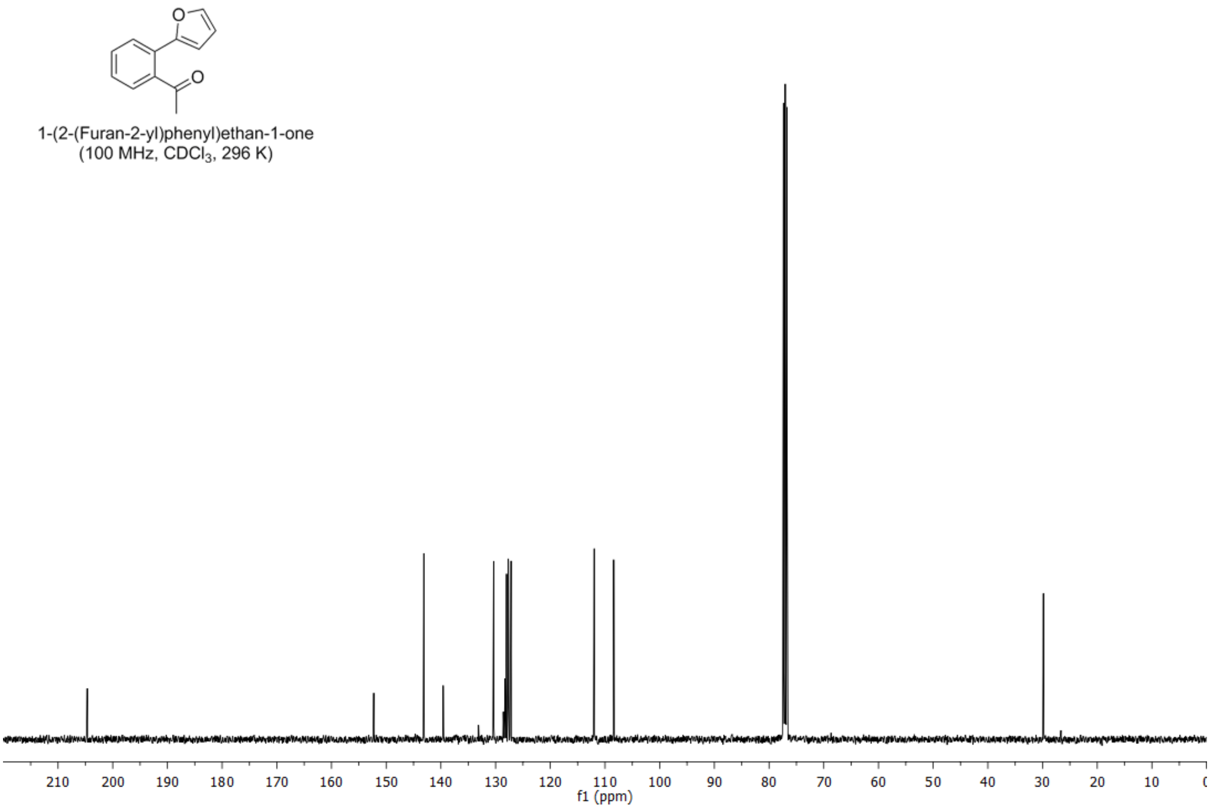

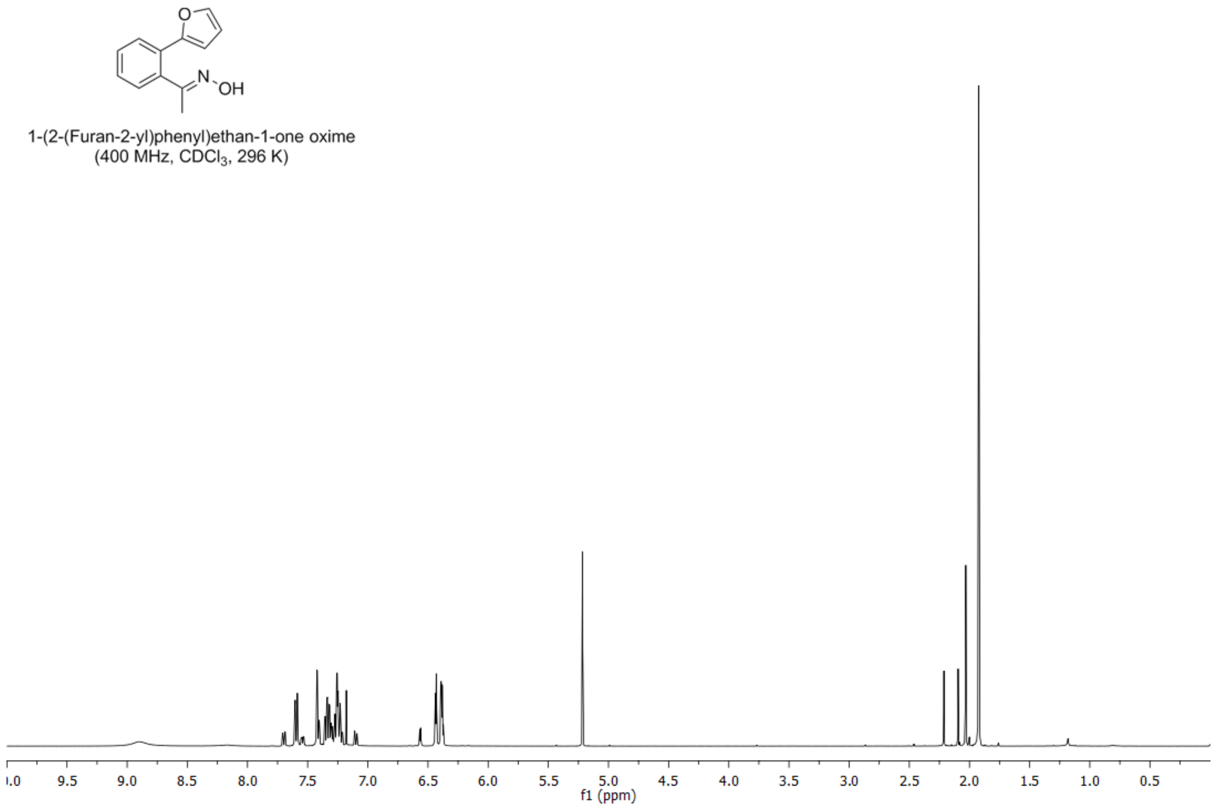
O
N
OH
1-(2-(Furan-2-yl)phenyl)ethan-1-one oxime
(400 MHz, $CDCl_3$, 296 K)
9.5 9.0 8.5 8.0 7.5 7.0 6.5 6.0 5.5 5.0 4.5 4.0 3.5 3.0 2.5 2.0 1.5 1.0 0.5
f1 (ppm)

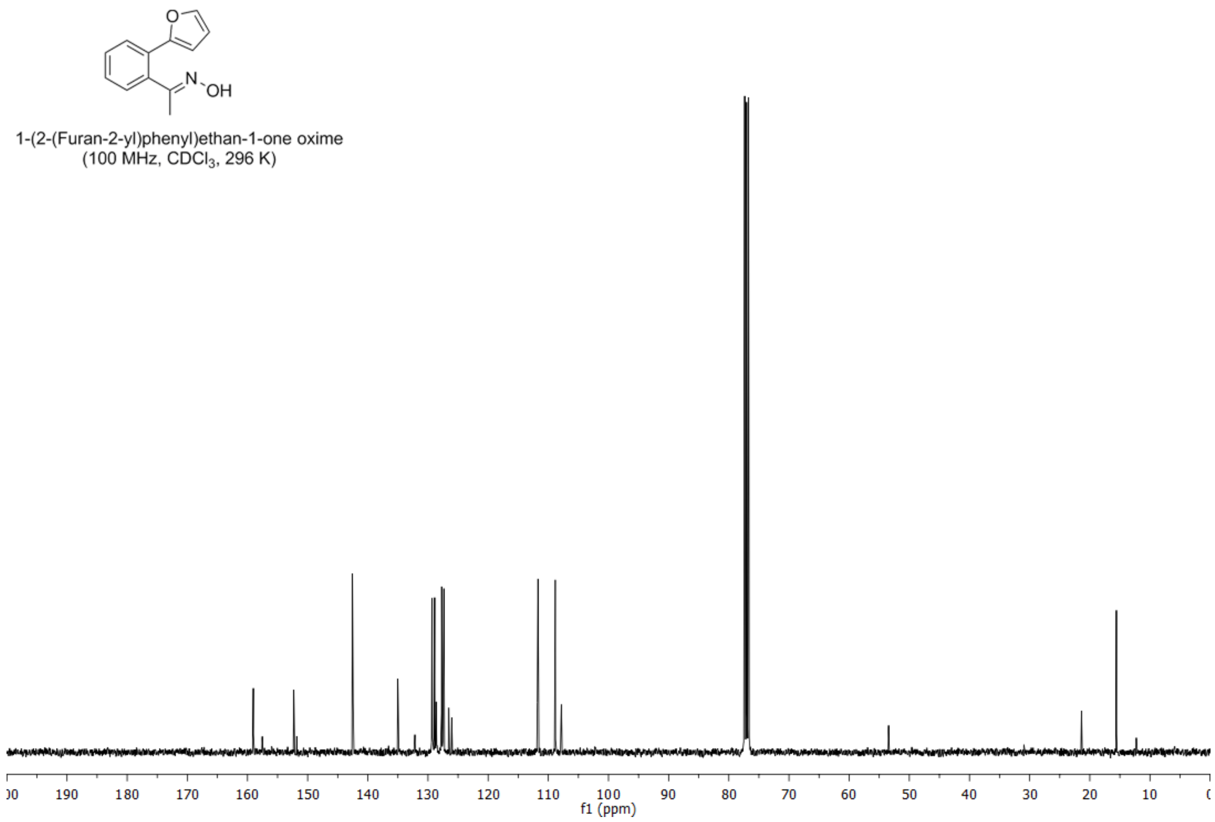
O
N
OH
1-(2-(Furan-2-yl)phenyl)ethan-1-one oxime
(100 MHz, $CDCl_3$, 296 K)
190 180 170 160 150 140 130 120 110 100 90 80 70 60 50 40 30 20 10
f1 (ppm)

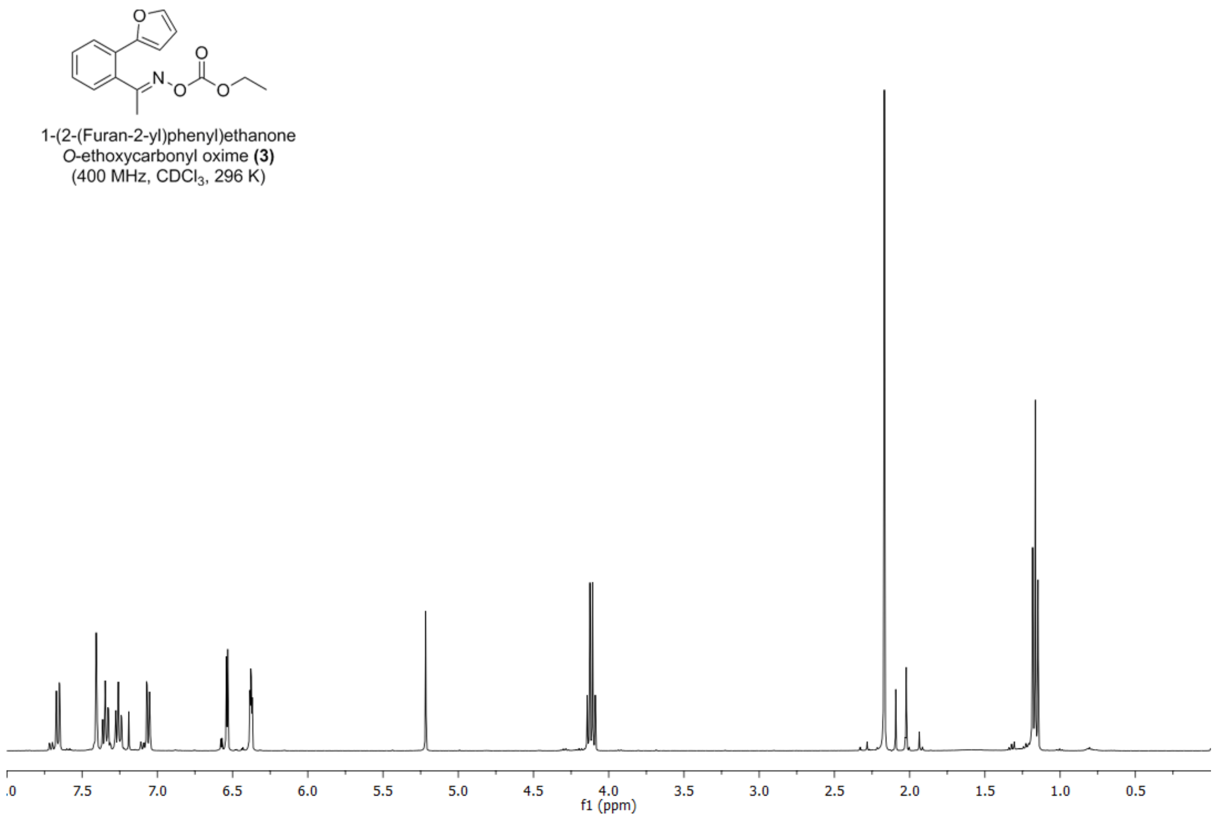
1-(2-(Furan-2-yl)phenyl)ethanone
O-ethoxycarbonyl oxime **(3)**
(400 MHz, $CDCl_3$, 296 K)
f1 (ppm)

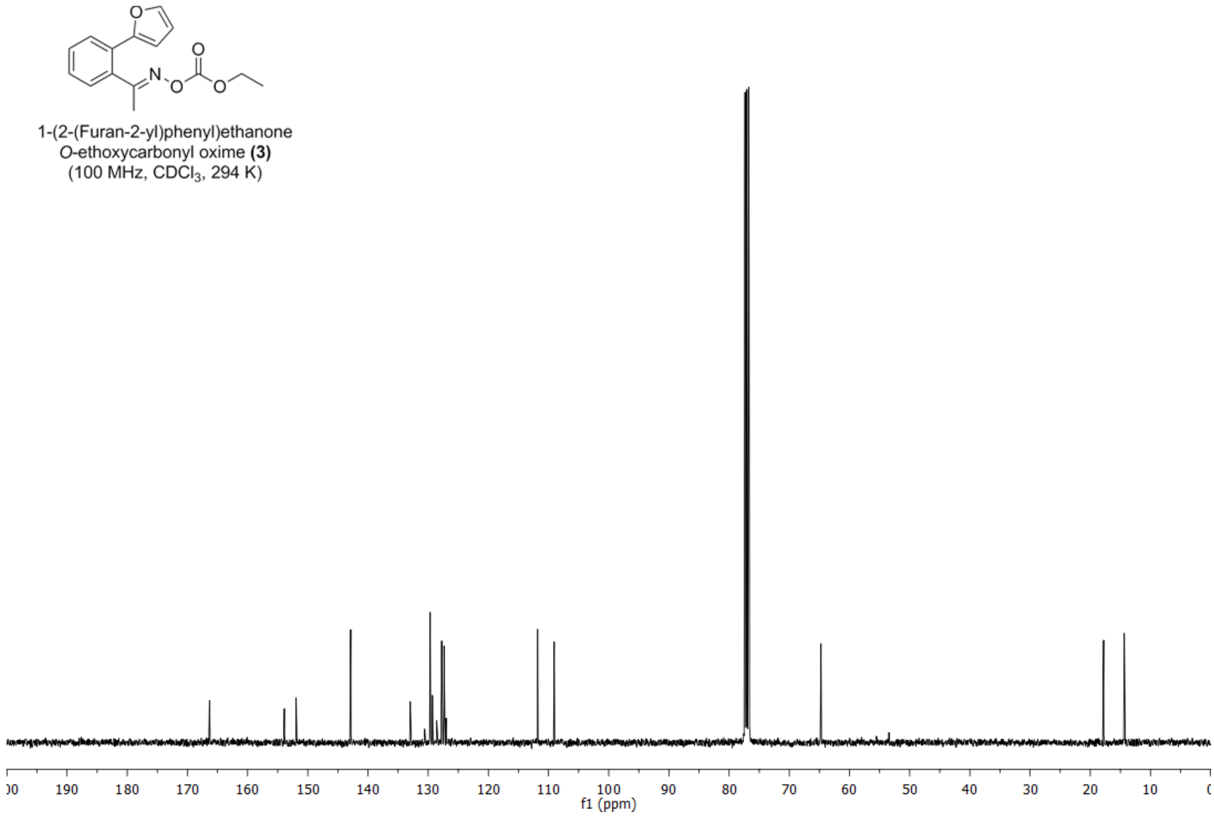
1-(2-(Furan-2-yl)phenyl)ethanone
O-ethoxycarbonyl oxime **(3)**
(100 MHz, $CDCl_3$, 294 K)
f1 (ppm)

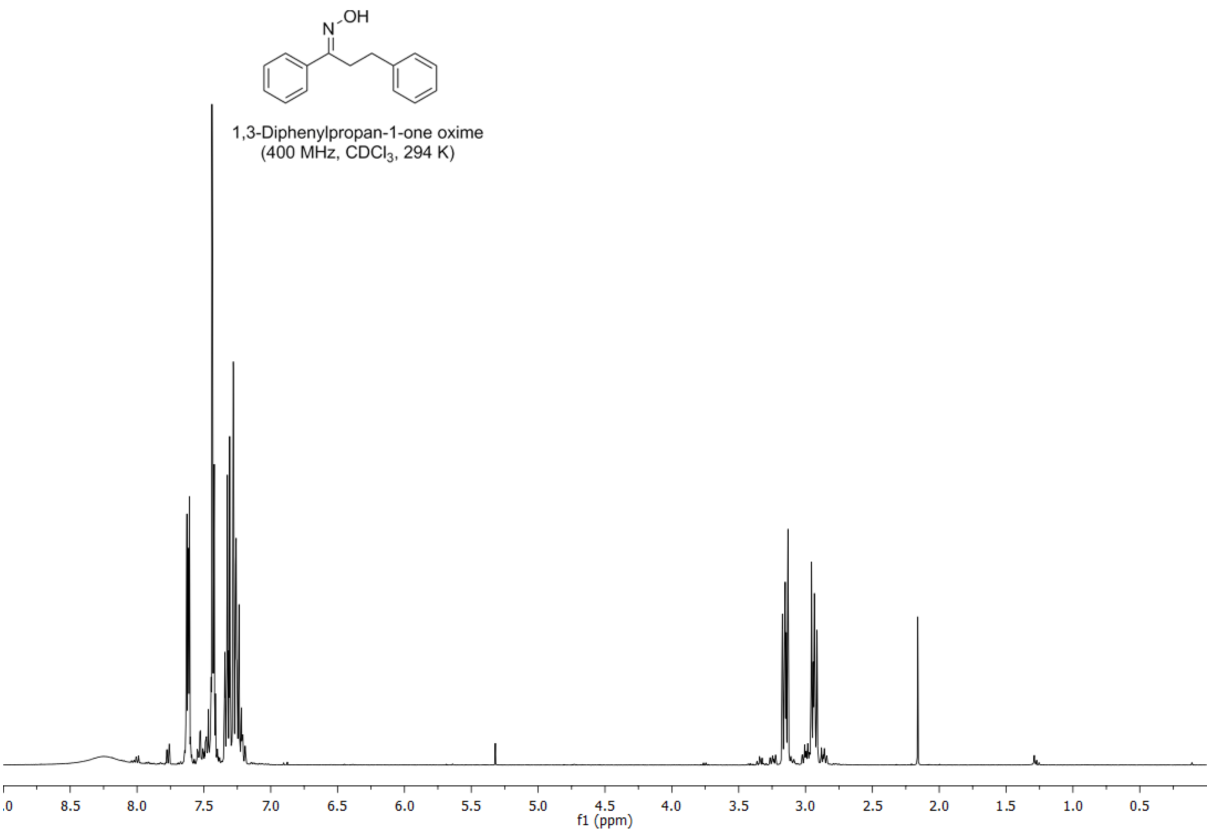
N
OH
1,3-Diphenylpropan-1-one oxime
(400 MHz, $CDCl_3$, 294 K)
f1 (ppm)

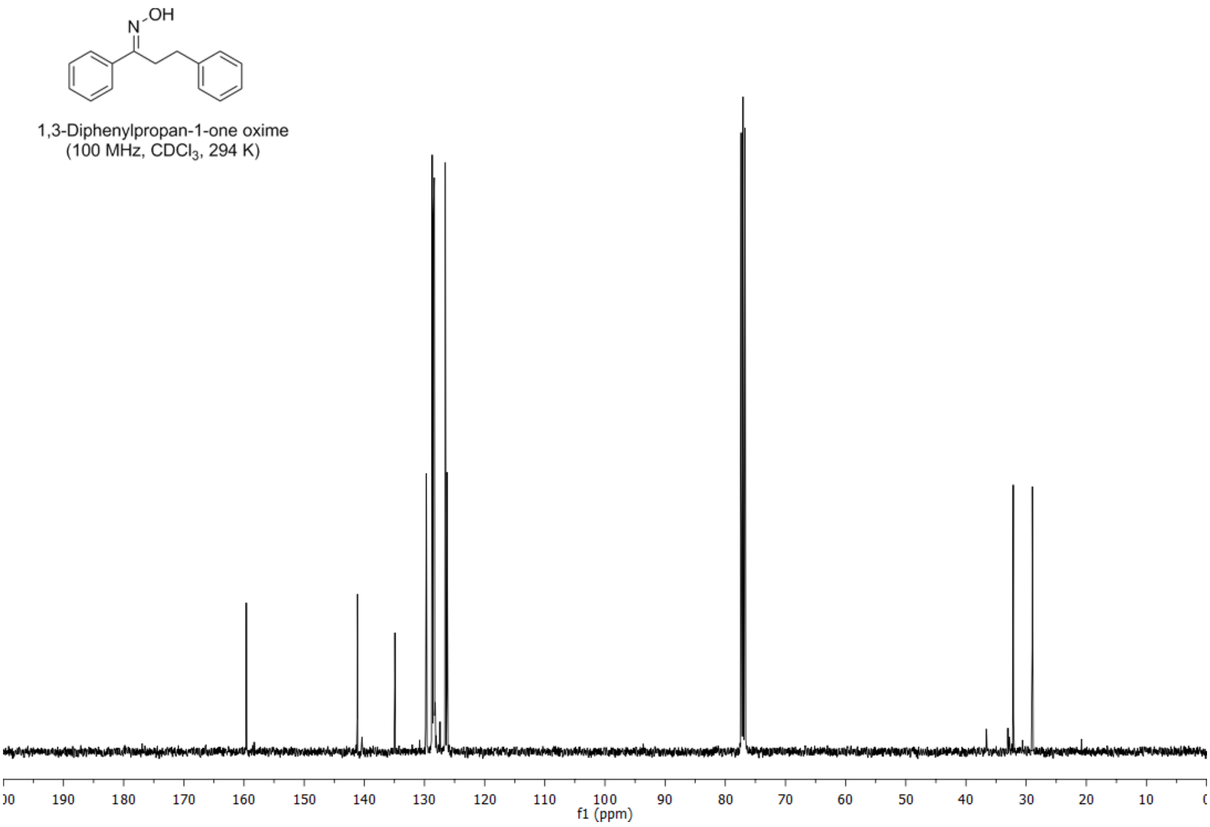
N
OH
1,3-Diphenylpropan-1-one oxime
(100 MHz, $CDCl_3$, 294 K)
f1 (ppm)

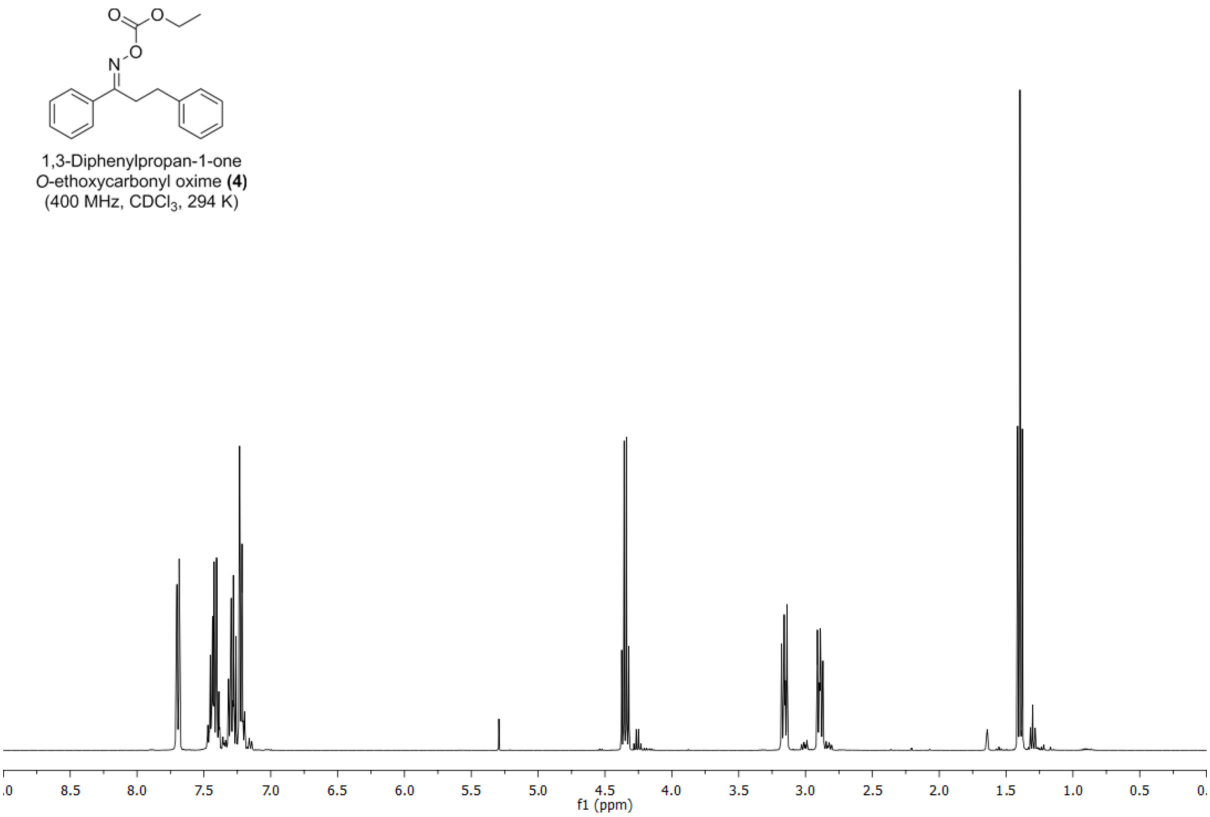
1,3-Diphenylpropan-1-one
O-ethoxycarbonyl oxime (4)
(400 MHz, CDCl3, 294 K)
f1 (ppm)

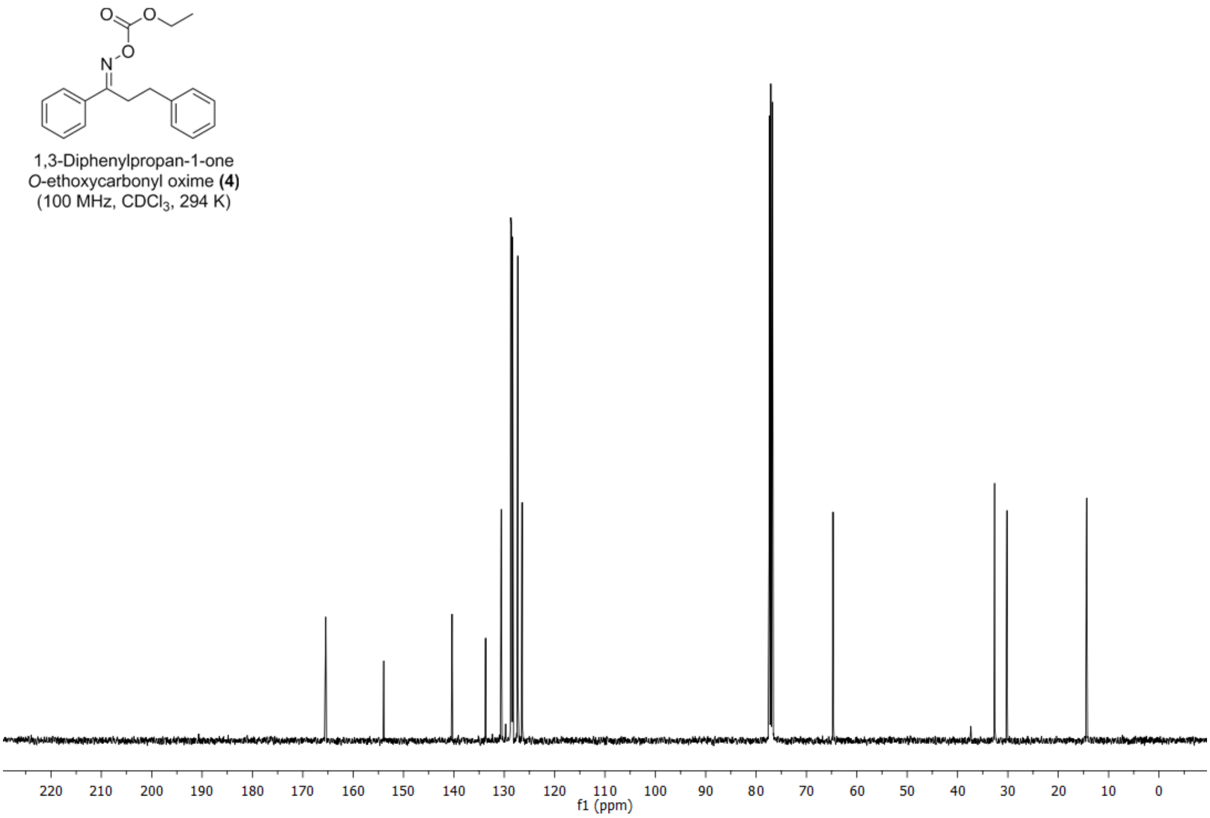
1,3-Diphenylpropan-1-one
O-ethoxycarbonyl oxime (4)
(100 MHz, CDCl3, 294 K)
f1 (ppm)
